# Computer vision-based wood identification and its expansion and contribution potentials in wood science: A review

**DOI:** 10.1186/s13007-021-00746-1

**Published:** 2021-04-28

**Authors:** Sung-Wook Hwang, Junji Sugiyama

**Affiliations:** 1 Graduate School of Agriculture, Kyoto University, Sakyo-ku, Kyoto, 606-8502 Japan; 2 College of Materials Science and Engineering, Nanjing Forestry University, Nanjing, 210037 China

**Keywords:** Convolutional neural networks, Computer vision, Deep learning, Image recognition, Machine learning, Wood identification, Wood anatomy

## Abstract

The remarkable developments in computer vision and machine learning have changed the methodologies of many scientific disciplines. They have also created a new research field in wood science called computer vision-based wood identification, which is making steady progress towards the goal of building automated wood identification systems to meet the needs of the wood industry and market. Nevertheless, computer vision-based wood identification is still only a small area in wood science and is still unfamiliar to many wood anatomists. To familiarize wood scientists with the artificial intelligence-assisted wood anatomy and engineering methods, we have reviewed the published mainstream studies that used or developed machine learning procedures. This review could help researchers understand computer vision and machine learning techniques for wood identification and choose appropriate techniques or strategies for their study objectives in wood science.

## Background

Every tree has clues that can help with its identification. Leaves, needles, barks, fruits, flowers, and twigs are important features for tree identification. However, most of these features are lost in harvested logs and processed lumber, so anatomical features are used as clues for wood identification. Fortunately, the International Association of Wood Anatomists (IAWA) has published lists of microscopic features for wood identification [1, 2]. These lists are the fruits of the work of wood anatomists and are well established, so they can be used with confidence to identify wood.

Conventional wood identification is performed by visual inspection of physical and anatomical features. In the field, wood identification is performed by observing macroscopic characteristics such as physical features, including color, figure, and luster, as well as macroscopic anatomical structures in cross sections, including size and arrangement of vessels, axial parenchyma cells, and rays [3]. In the laboratory, wood identification is performed by observing various anatomical features microscopically from thin sections cut in three orthogonal directions, cross, radial, and tangential [4]. Wood identification is a demanding task that requires specialized anatomical knowledge because there are huge numbers of tree species, as well as various patterns of inter-species variations and intra-species similarities. Therefore, visual inspection-based identification can result in misidentification by the wrong judgment of a worker. Unsurprisingly, this is a major problem at the forefront of industries where large quantities of wood must be identified within a limited time.

The spread of personal computers has triggered a major turning point in wood identification. Wood anatomists have created a new system called computer-assisted wood identification [5] by computerizing the existing card key system [6, 7]. Several computerized key databases and programs have been developed to take advantage of the new system [8–10]. Because of the vast biodiversity of wood, the deployed databases generally cover only those species that are native to a country or a specific climatic zone [8, 9, 11, 12]. Although this system has made the identification of uncommon woods easier, traditional visual inspection was preferred for efficiency reasons in the identification of commercial woods [3]. The computer-aided wood identification systems used explicit programming, which required the user to program all the ways in which the software can work. That is, the user had to teach the software all the identification rules, which was never an efficient way because there are so many rules for wood identification. This programmatic nature made it difficult to spread the system globally.

Over time, computer-aided wood identification settled with web-based references such as ‘Inside Wood’ at North Carolina State University [13] and ‘Microscopic identification of Japanese woods’ at Forestry and Forest Products Research Institute (FFPRI), Japan [14]. These are very useful open wood identification systems that cover a wide variety of woods, but require expert knowledge of wood anatomy. As such, there are various obstacles to the further development of computer-aided wood identification, so this is where machine learning (ML) comes in.

ML is a type of artificial intelligence (AI) where a system can learn and decide exactly what to do from input data alone using predesigned algorithms that do not require explicit instructions from a human [15, 16]. In a well-designed ML model, users no longer have to teach the model the rules for identifying wood, and even wood anatomists are not required to find wood features that are important for identification. Computer vision (CV) is a computer-based system that detects information from images and extracts features that are considered important [17–19]. Automated wood identification that combines CV and ML is called computer vision-based wood identification [20, 21]. AI systems based on CV and ML are making great strides in general image classification [22–25]. The same is true for wood identification and related studies have been increasing [20, 26–29].

Wood identification is a major concern for tropical countries with abundant forest resources, so there is a high demand for novel wood identification systems to address the wide biodiversity. There are various on-site needs for wood identification, such as preserving endangered species, regulating the trade of illegally harvested timbers, and screening for fraudulent species [30–33]. However, it is practically impossible to train a sufficient number of field identification workers to meet the demands of the field. Wood identification requires expert knowledge of wood anatomy and long experience, so even if a lot of money and time is spent, there are practical limits to the training of skilled workers [34].

To answer the demands in the field, various approaches have been proposed, such as mass spectrometry [35–37], near-infrared spectroscopy [38, 39], stable isotopes [40, 41], and DNA-based methods [42, 43]. However, these approaches have practical limitations as a tool to assist or replace the visual inspection due to their relatively high cost and procedural complexity. This is where CV-based identification techniques and ML models can be very important. Clearly, automated wood identification systems are urgently needed and CV-based wood identification has emerged as a promising system.

In this review, we provide an overview of CV-based wood identification from studies reported to date. CV techniques used in other contexts, such as wood grading, quality evaluation, and defect detection, are outside the scope of this review. This review covers CV-based identification procedures, provides key findings from each process, and introduces the emerging interests in CV-based wood anatomy.

## Workflow of CV-based wood identification systems

Image recognition or classification is a major domain in AI and is generally based on supervised learning. Supervised learning is a ML technique that uses a pair of images and its label as input data [44]. That is, the classification model learns labeled images to determine classification rules, and then classifies the query data based on the rules. Conversely, in unsupervised learning the model itself discovers unknown information by learning unlabeled data [45]. Classification is generally a task of supervised learning and clustering is generally a task of unsupervised learning.

**Fig. 1:**
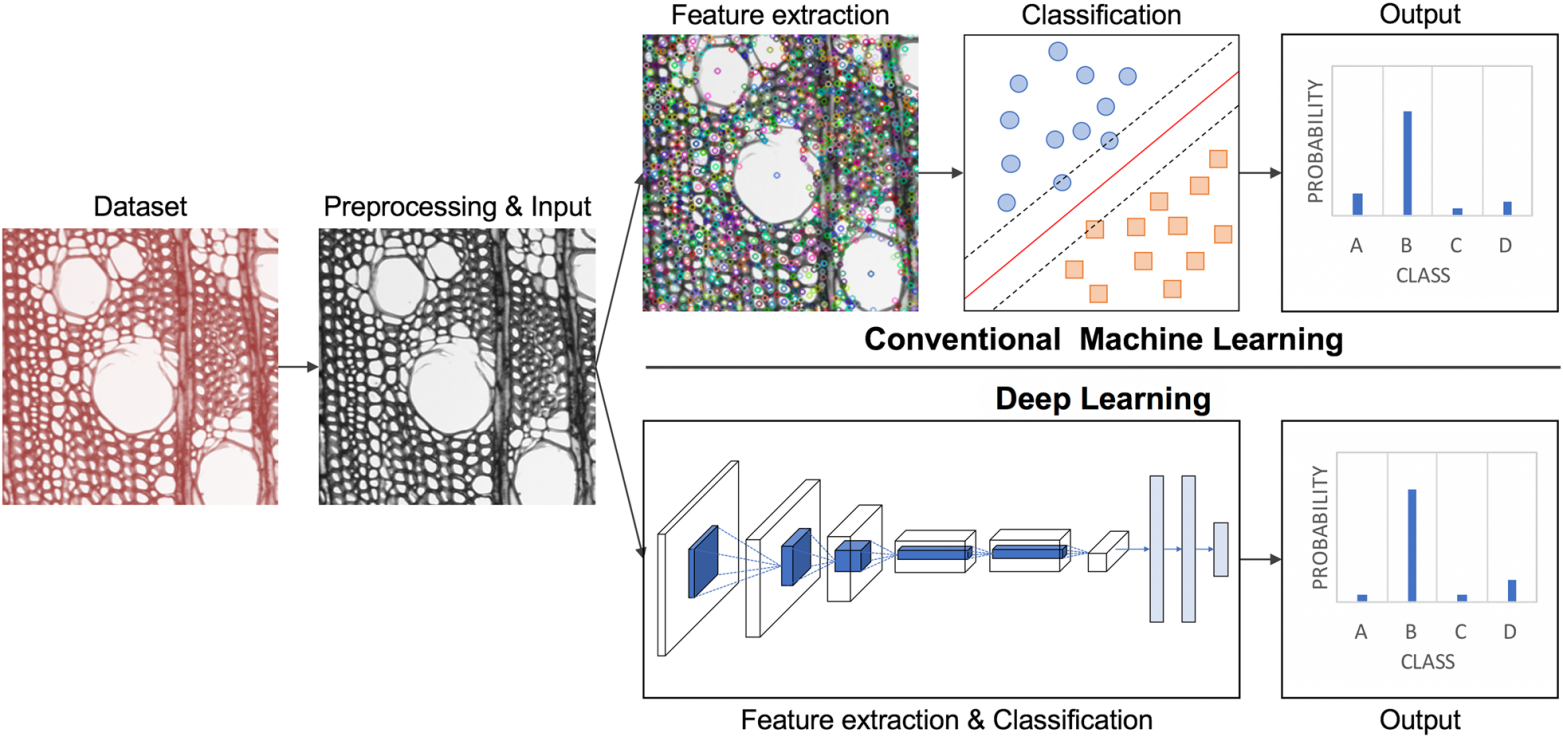
General workflow of conventional machine learning and deep learning models for image classification

CV-based wood identification systems follow the general workflow presented in Fig. 1. Image classification is divided methodologically into conventional ML and deep learning (DL), both of which are forms of AI. In conventional ML, feature extraction, the process of extracting important features from images (also called feature engineering), and classification, the process of learning the extracted features and classifying query images, are performed independently. First, all the images in a dataset are preprocessed using various image processing techniques to convert them into a form that can be used by a particular algorithm to extract features. Then, the dataset is separated into training and test sets, and the features are extracted from the training set images using feature extraction algorithms. A classifier establishes the classification model by learning the extracted features. Finally, the test set images are input and the classification model, which returns the predicted classes of each image, thus completing the identification.

In DL, feature extraction and classification are performed in one integrated process [21, 29], which is end-to-end learning using annotated images [46]. The feature learning process using feature engineering techniques in conventional ML usually allows manual intervention by the user but manual intervention is limited in DL. Subsequent sections describe the process from image acquisition to classification, following the workflow of CV-based wood identification.

## Image databases

### Image acquisition

CV-based wood identification starts with image acquisition. It takes a considerable amount of time and effort to get enough wood samples to build a new dataset. For this reason, most studies have used Xylarium collections [26, 47–52]. Most studies only captured cross-sectional images of wood blocks except for a few studies using lumber surface [53] or three orthogonal sections [54, 55]. The surfaces of the blocks are cut with a knife or sanded with sandpapers to clearly reveal the anatomical characteristics. Macroscale images can be captured directly from the wood blocks using a digital camera or stereo microscope. To capture microscale images, meanwhile, microscope slides of wood samples must be prepared through standard procedures leading to softening, cutting, staining, dehydration, and mounting [56].

In image capturing, the quality of the obtained images can vary depending on lighting conditions. Imaging modules equipped with optical systems have been used to control the lighting uniformly [21, 51, 57], and image processing techniques such as filtering were applied to normalize the brightness of the captured images [58–61]. Digital image processing is to be covered in section preprocessing.

### Image type

**Fig. 2:**
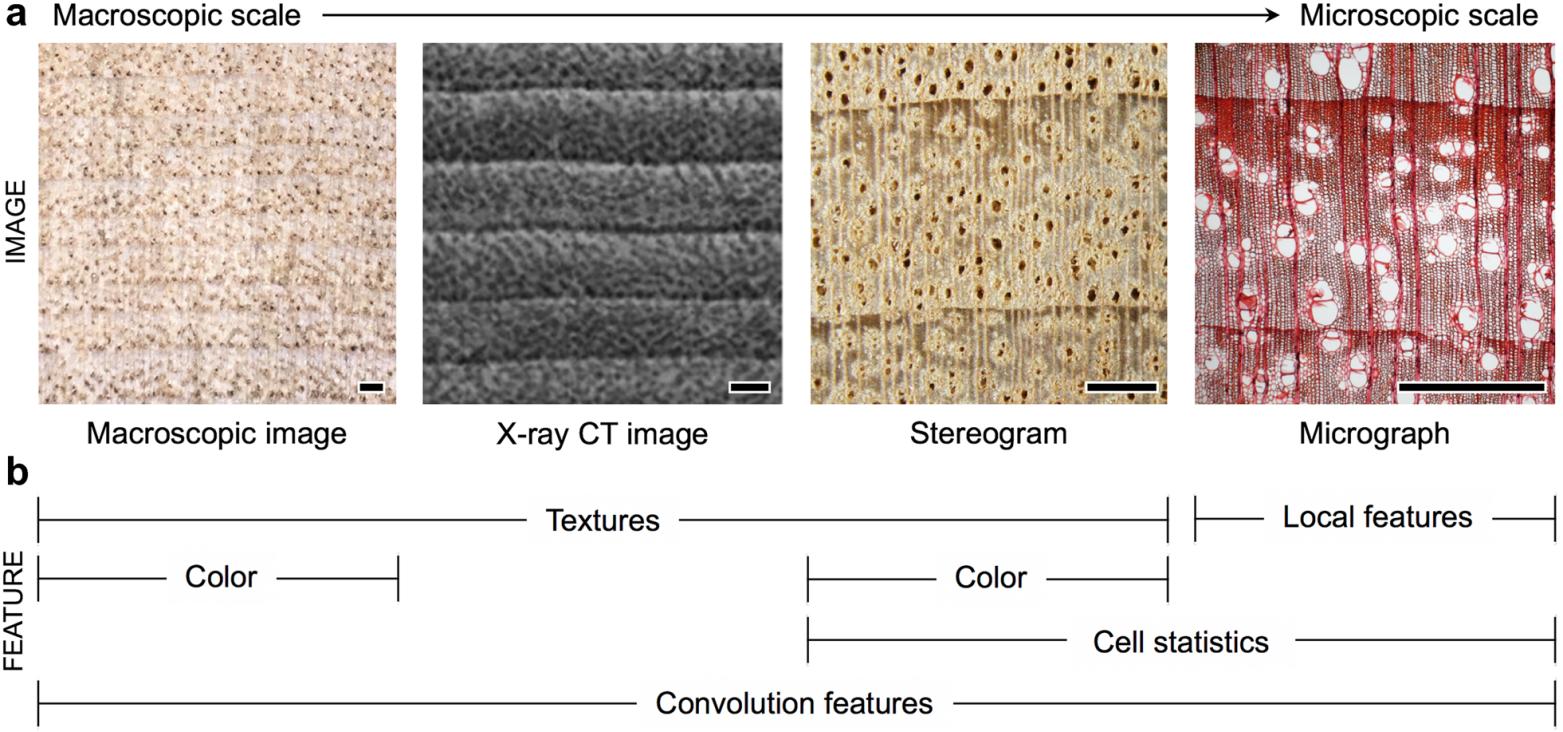
Image types obtainable from wood and the corresponding extractable image features. **a** Macroscopic image, X-ray computed tomographic (CT) image, stereogram, and micrograph of cross sections of *Cinnamomum camphora*. **b** Preferred features by image type in publications. Scale bars = 1 mm

All wood image types can be used as data for identification. The most commonly used image types are macroscopic images [50, 54, 62–65], X-ray computed tomographic (CT) images [66, 67], stereograms [20, 21, 26, 68, 69], and micrographs [47, 70–72] (Fig. 2a). Macroscopic images are images taken without magnification by a regular digital camera. Stereograms are generally images taken at the hand lens magnification (10×), but higher magnifications may be used depending on the purpose. Micrographs are optical microscopic images and they are commonly used in conventional wood identification. X-ray CT images are a slice of the original images generated by X-ray CT scans.

Macroscopic images and stereograms were preferred in studies aimed at developing field-deployable systems because they were easily obtained only by smoothing the wood surface [49, 51, 57, 70]. Microscopic level features extracted from micrographs allow for an anatomical approach because the image scale is the same as that used in established wood anatomy [48, 73, 74]. X-ray CT images have been used to identify wooden objects with limited sampling, such as registered cultural properties, because of the non-destructive nature of the imaging [66, 67]. In one study, the morphological features of wood cells were extracted from scanning electron microscope images [75].

The extractable or effective image features that are used for wood identification can differ depending on the image type (Fig. 2b), so the most suitable image type for the research purpose and for the wood should be considered carefully. As shown in Fig. 2a, in macroscale images, macroscopic images, and X-ray CT images, large wood cells and cell aggregates such as annual rings, rays, and vessels are observed from the cross-sectional image. Stereograms provide more detailed anatomical characteristics, such as the type of vessel and axial parenchyma cell. Anatomical characteristics observed from the macroscale images are treated as a matter of texture classification (Fig. 2b) because they are represented as the spatial distribution of intensity between adjacent pixels and repetitive patterns [76]. Because macroscopic images and stereograms retain the unique color of wood, the color information was used for wood identification [58, 62, 63, 77, 78]. In micrographs that provide microscopic information such as wood fibers, local features for extracting morphological characteristics of cells were preferred [48, 74, 79], and statistics related to the size, shape, and distribution of cells were also used for identification [27, 59, 61, 80]. In contrast, convolutional neural networks (CNNs) were employed regardless of image type [29, 65, 72, 81].

### Databases

A good identification ML model can be built only from a good reference image collection. Wood image data should contain the specific features of each species and provide sufficient scale for the features to be observed. A quantitatively rich database is required so that the ML model can learn the various biological variations that occur within a species.

Published wood image databases constructed for CV-based wood identification are listed in Table 1. CAIRO and FRIM, which contain stereogram images of commercial hardwood species in Malaysia, were among the first to be constructed and are still regularly updated [27, 82]. LignoIndo, which also contains stereogram images, was constructed for the development of a portable wood identification system [83]. It contains images of Indonesian commercial hardwood species.

**Table 1:** Published wood image databases that have been used in CV-based wood identification studies

Database	Description	Image type	#SP/#IMG	Accessibility	Ref.
CAIRO	Commercial hardwood species in Malaysia	Stereo	37/3700	Inaccessible	[82]
FRIM	Commercial hardwood species in Malaysia	Stereo	52/5200	Inaccessible	[27]
LignoIndo	Commercial hardwood species in Indonesia	Stereo	809/4854	Inaccessible	[83]
ZAFU WS 24	Wood species in Zhejiang A&F University	Stereo	24/480	Inaccessible	[68]
RMCA	Commercial wood species in Central-Africa	Micro	77/1221	Open	[86]
XDD	Major Fagaceae species in Japan	Micro	18/2449	Open	[88]
	Lauraceae species in East Asia	Micro	39/1658	Open	[89]
WOOD-AUTH	Wood species in Greece	Macro	12/4272	Open	[87]
UFPR	Wood species in Brazil	Macro	41/2942	Open	[84]
		Micro	112/2240	Open	[85]

*Stereo* stereogram, *Micro* micrograph, *Macro* macroscopic image, *#SP* number of species, *#IMG* number of images, *Ref.* reference

UFPR, an open image database of Brazilian wood, contains macroscopic images and micrograph datasets. It was established to serve as a benchmark for automated wood identification studies and is accessible from the Federal University of Paraná website [84, 85]. Other publicly available wood image databases are RMCA, which contains micrograph images of Central-African commercial wood species [86], WOOD-AUTH, which contains macroscopic images of Greek wood [87], and the Xylarium Digital Database (XDD), which contains micrograph-based multiple datasets [88, 89] (Table 1). To measure or improve the performances of wood identification models, benchmarks for performance evaluation are essential. These open databases have contributed to the development of CV-based wood identification.

All the databases contain only cross-sectional images, regardless of image type. Barmpoutis et al. [54] compared the discriminative power of three orthogonal sections of wood and reported that the model trained with the cross-sectional image dataset had a higher classification performance than the same model trained with other sections or combinations of them.

### Xylarium digital database for wood information science and education

The biggest obstacle currently faced by CV-based wood identification is the absence of large databases. The development of the ImageNet dataset, which contains 14.2 million images across more than 20,000 classes, ended the AI winter [90]. Similarly, large databases are essential for progressing CV-based wood identification. Historically, the construction of large image databases for wood science has always been a challenge [6, 81], mainly because wood images are cumbersome to make and only wood anatomists can annotate the images correctly. Hence, their construction requires extensive collaboration across many organizations in wood science.

A first step in constructing a large database could be the digitization of Xylaria data that is distributed around the world, accompanied by the establishment of standard protocols for image data generation [91]. Once digitization is complete, data sharing and/or integration systems would need to be discussed. Unlike the Xylaria data, a digital herbaria dataset that covers a wide range of biological diversity has already been established [92].

Under these circumstances, the recently released Xylarium Digital Database (XDD) for wood information science and education is notable. XDD is a digitized database based on the wood collection of the Kyoto University Xylarium database. It contains 16 micrograph datasets, covering the widest biological diversity among open digital databases released to date (Table 2). The datasets in XDD have been used in multiple studies [48, 73, 74, 93], and based on the findings of these studies, each wood family was divided into two datasets with two different pixel resolutions. A large digital database built by combining individual databases such as XDD will be an important contribution to the advancement of wood science with state-of-the-art DL techniques beyond CV-based wood identification.

**Table 2:** Datasets in the Xylarium Digital Database (XDD) for wood information science and education

Dataset	Family	Number of				Resolution (µm/pixel)	DOI
		Genus	Species	Individual	Image		
XDD_001	Betulaceae	5	19	70	817	4.44	10.14989/XDD_001
XDD_002	Betulaceae	5	19	70	817	2.96	10.14989/XDD_002
XDD_003	Cannabaceae	3	3	23	317	4.44	10.14989/XDD_003
XDD_004	Cannabaceae	3	3	23	317	2.96	10.14989/XDD_004
XDD_005	Fagaceae	5	18	185	2446	4.44	10.14989/XDD_005
XDD_006	Fagaceae	5	18	185	2446	2.96	10.14989/XDD_006
XDD_007	Lauraceae	11	39	131	1658	4.44	10.14989/XDD_007
XDD_008	Lauraceae	11	39	131	1658	2.96	10.14989/XDD_008
XDD_009	Magnoliaceae	2	18	37	926	4.44	10.14989/XDD_009
XDD_010	Magnoliaceae	2	18	37	926	2.96	10.14989/XDD_010
XDD_011	Sapindaceae	5	18	56	444	4.44	10.14989/XDD_011
XDD_012	Sapindaceae	5	18	56	444	2.96	10.14989/XDD_012
XDD_013	Ulmaceae	2	4	38	443	4.44	10.14989/XDD_013
XDD_014	Ulmaceae	2	4	38	443	2.96	10.14989/XDD_014
XDD_015	Hardwood^a^	33	119	540	7051	4.44	10.14989/XDD_015
XDD_016	Hardwood^a^	33	119	540	7051	2.96	10.14989/XDD_016

The images that make up the datasets are optical micrographs that correspond to an actual area of 2.7 × 2.7 mm^2^. Image resolutions of 4.44 and 2.98 µm/pixel correspond to image sizes of 600 × 600 and 900 × 900 pixels, respectively

^a^Dataset with seven classes integrated per resolution

## How computer vision processes images

**Fig. 3:**
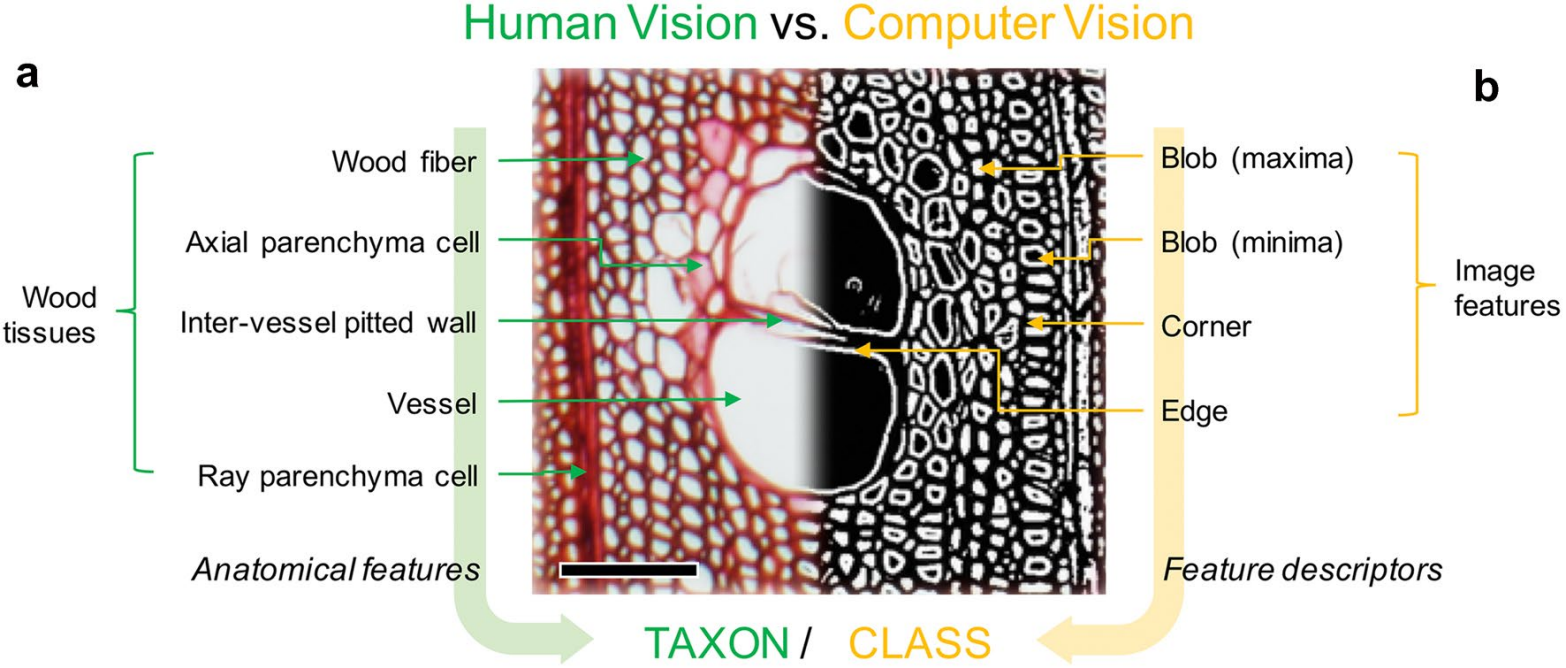
Features observed by human vision and extracted by computer vision for wood identification. **a** Cross-sectional optical microscopic image of *Cinnamomum camphora*. **b** Difference-of-Gaussian image, which presents the other half of the optical microscopic image in **a**. Scale bar = 100 µm

To identify woods, wood anatomists observe the anatomical characteristics of wood tissues such as wood fibers, axial parenchyma cells, vessels, and rays, as well as their size and arrangement from wood sections (Fig. 3a), whereas CV is used to extract features such as points, blobs, corners, and edges, and their patterns from images (Fig. 3b).

**Fig. 4:**
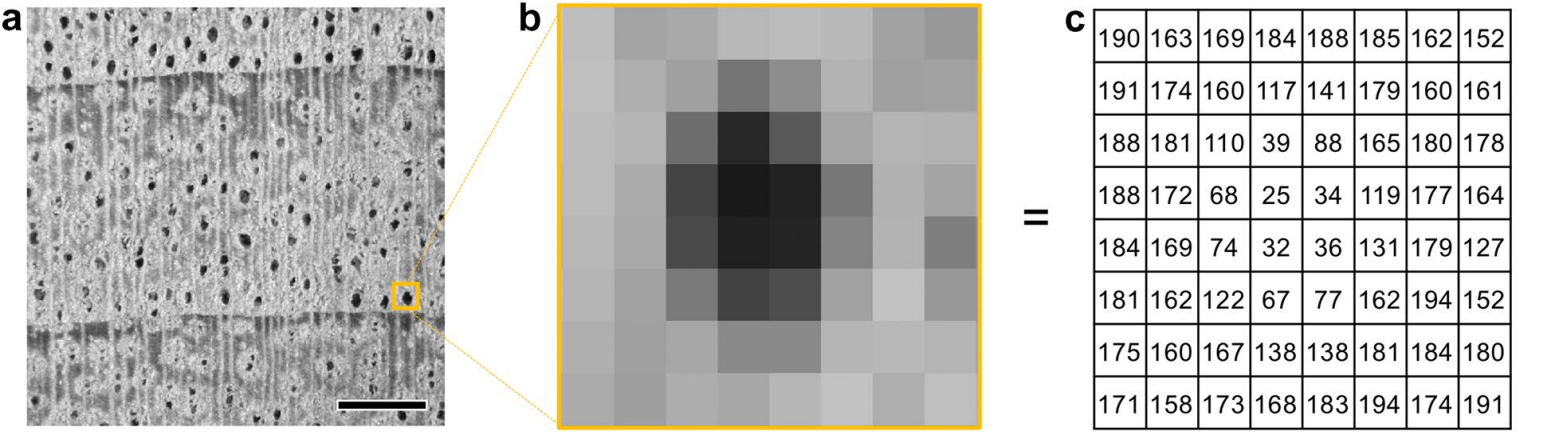
Grayscale image expressed in a matrix of numbers that computers can process. **a** 8-bit grayscale stereogram of a cross section of *Cinnamomum camphora*. **b** Enlarged image of the yellow box in **a**, which contains a vessel. **c** Gray values of each pixel in **b**. Scale bar = 1 mm

For computers, an image is a combination of many pixels and is recognized as a matrix of numbers with each number representing a pixel intensity (Fig. 4). Wood images are made up of various cell types. Different wood species have different patterns of anatomical elements, and the composition of the elements causes distinctions in pixel intensity, arrangement, distribution, and aggregation patterns. Such differences are detected by CV and learned by ML. This is the fundamental concept of CV-based wood identification.

## Preprocessing

Image preprocessing is a preliminary step of feature extraction that facilitates extraction of predefined features and reduces computational complexity [94]. Various image preprocessing techniques have been used depending on the problem to be solved.

Simple tasks such as grayscale conversion and image cropping and scaling are preprocessing techniques commonly used in the conventional ML models [26–28, 52, 62, 71, 95]. The color of wood generally is not regarded as important information in because it is easily changed by various factors. In conventional ML RGB color images are converted to grayscale images, which provide enough information to recognize species specificity and also significantly reduce computational costs. Whereas, DL models use RGB images without conversion [29, 65, 81, 96].

Cropping is the process of extracting parts of the original image to remove unnecessary areas or to focus on specific areas [29, 65, 96–99]. Scaling is the process of changing the image size in relation to pixel resolution. High-resolution images have excessive computational costs [100], so the image size needs to be adjusted but remain within the range in which the expected features can be extracted. Information loss should be considered when resizing wood images, and information distortions inevitably occur when changing aspect ratios.

Homomorphic filtering is a generalized technique for image processing and commonly used for correcting non-uniform illumination. This technique is preferred to normalize the brightness across an image and increase contrast in wood identification [58–61]. Homomorphic filtering also has the effect of sharpening the image [26, 101] and Gabor filters have been used for sharpening [102]. Sharpening was used as a preprocessing to segment notable cells such as the vessel [59, 61, 103].

Denoising using a median or adaptive filter has been performed to remove noise or artifacts in images [75, 99, 104], and equalization of the gray level histogram was shown to improve the contrast [26, 75, 101]. For motion blurred images, deconvolution using the Richardson–Lucy algorithm was effective for deblurring [105]. For X-ray CT images, pixel intensities are directly related to wood density, so gray level calibration based on wood density is an important process for predicting physical and mechanical properties as well as for wood identification [67]. By preprocessing images from various sources, image data can be cleaned up and standardized, which reduces data complexity and improves algorithm accuracy.

## Data splitting

When a wood image dataset has been processed and is ready for use, the next step is to split it into subsets. One of the goals of ML is to build a model with high prediction performance for unseen data [106]. Non-split, that is training data only, and two-split into training and test sets can result in poor prediction for unseen data because they build models that fit best only on anatomical features of training and test data, respectively. Therefore, the most common splitting method is to split the data into training, validation, and test sets.

The training set is used to construct a classification model. The classifier learns the features extracted from the training set images and their labels to build a classification model. The validation set is used to optimize the training set by tuning the parameters during model building. The validation set can be specified as an independent set or a part of the training set can be iteratively selected, such as *k*-fold cross-validation [107]. Although this method is standard in conventional ML, it is not often used when training a large model in DL because the training itself has a large computational scale. The test set is used to evaluate the performance of the final model built by learning the training set.

The split ratio for a dataset depends on the data. Splitting training and test data sets 8-to-2 was preferred and this can be a good start. Guyon [108] suggested that the validation set should be inversely proportional to the square root of the number of free adjustable parameters, but it should be noted that there is no ideal ratio for splitting a dataset. It is important to find a balance between training and test sets because a small training set can result in high variance of parameter estimates and a small test set can result in high deviations in performance statistics.

In general classification problems, random dataset splitting is considered a good approach [109], but for biological image data such as wood images, especially microscopic scale images, it is not ideal. If multiple images obtained from an individual are divided into training and test sets, the classification model can correctly classify the test images because the model has already learned the characteristics of the individual from the training set, even if the images represent different areas of the sample. In such a case, the classification performance of the model will be high, but this result is caused by overfitting and does not guarantee the generalization performance of the model [44]. In CV-based wood identification, therefore, it is desirable to split a dataset by individual units, not by images, because the splitting process is important in determining the reliability of classification models. Many published studies used random splitting methods [29, 61, 66, 68, 79], but many studies did not provide details of the dataset splitting scheme.

## Conventional machine learning

### Feature extraction

Image features are the information that is required to perform specific tasks related to CV applications. In general, the features refer to local structures such as points, corners, and edges, and global structures such as patterns, objects, and colors in an image. CV uses a diverse collection of feature extraction algorithms. In wood identification based on conventional ML, different feature types are selected depending on the type of image and classification problem, most of them are for texture and local features.

#### Texture feature

Texture is the visual pattern of an image and it is expressed by the combination and arrangement of image elements. That is, texture is information about the spatial arrangement of pixel intensities in an image, and this information is quantified to obtain the texture feature. Early studies generally approached wood identification as a matter of texture classification because cross sections of wood represent different anatomical arrangements, namely different patterns, depending on the species [20, 26, 82, 101, 110]. Textures are the image features that were most preferred in published studies, and this is closely related to the finding that stereograms and macroscopic images were preferred for developing field-deployable systems [21, 49, 57, 69].

Gray level co-occurrence matrix (GLCM) is a statistical approach for determining the texture of an image by considering the spatial relationship of pixel pairs, and GLCM is the classic and most widely used texture feature in wood identification [20, 26, 28, 66, 69, 98]. The GLCM feature is quantified by statistical methods such as the Haralick texture feature [111–113] that takes into account the direction and distance of the co-occurrences of two adjacent pixels. In the early days of CV-based wood identification, Tou et al. [20] reported 72% identification accuracy using five GLCM texture features extracted from 50 images of five tropical wood species. Subsequently, they scaled up their datasets (CAIRO) and studied various GLCM-based identification strategies such as rotation invariant GLCM [110] and multiple feature combinations [62, 114–116]. GLCM is a feature that has high computational cost, so one-dimensional GLCM [117] and image blocking have been considered for computational efficiency [97, 98]. Kobayashi et al. [66, 67, 99] constructed identification models trained with GLCM features extracted from X-ray CT images and stereograms and demonstrated that GLCM-based methods were promising for the identification of wood cultural properties.

To reduce the computational cost and improve the classification performance, the basic gray level aura matrix (BGLAM) was proposed [118]. This is the basis of the gray level aura matrix (GLAM) [119] developed to overcome the limitation of GLCM that it cannot contain information about the interaction between gray level sets in textures with large scale structure [120]. BGLAM is characterized by the co-occurrence probability distribution of gray levels in all possible displacement configurations and it has been actively used for wood identification [27, 28, 59, 61]. Zamri et al. [60] classified 52 species in the FRIM database using the improved-BGLAM algorithm, which realizes feature dimension reduction and rotational invariance from BGLAM, and the classification performance of their model using the improved-BGLAM far exceeded that using GLCM.

Local binary pattern (LBP) is a simple but efficient visual descriptor for representing image texture. LBP calculates the local texture of an image by comparing the value of a center pixel with those of the surrounding pixels in the grayscale image [121]. Nasirzadeh et al. [82] compared the performance of models trained with LBP-based features extracted from the CAIRO database and found that the LBP histogram Fourier features outperformed the conventional rotation-invariant LBP. Martins et al. [47] published their micrograph-based UFPR database and reported a 79% recognition rate for a classification model trained with LBP. In the same study, they also showed that when the original image was divided into sub-regions, the recognition rate of the model trained with LBP improved by 86%.

In comparative studies of GLCM and LBP, classification models trained with LBP always achieved better performances than those trained with GLCM for all image types (Table 3). While GLCM quantifies an image with only one value per texture feature, LBP represents an image with a 256-bin histogram, although it contains many overlapping patterns. Corners and Harlow [112] demonstrated that among the 14 measures proposed by Haralick et al. [111] for GLCM texture feature extraction, only five of them, energy, entropy, correlation, local homogeneity, and contrast, were sufficient for texture classification. That is, GLCM required a lower dimension of feature vectors for texture description than LBP. In addition, LBP histograms can identify local patterns such as edges, flats, and corners, which may explain why LBP outperformed GLCM in wood identification. It has been pointed out that it may be difficult to describe large wood cells, such as vessels and resin canals using LBP [95], but this problem can be solved by adjusting the radius of the LBP unit and combining units with different radii.

**Table 3:** Performances of GLCM, LBP, and LPQ texture features for wood identification

References	Database	Image type	#SP/#IMG	Classifier	Classification rate (%)		
					GLCM	LBP	LPQ
Prasetiyo et al. [147]	CAIRO	Stereo	25/2390	ANN	89.6	93.6	–
Martins et al. [47]	UFPR	Micro	112/2240	SVM	55.3	79.3	–
Kobayashi et al. [66]	–	XCT	6/240	*k*-NN	98.3	99.5	–
Cavalin et al. [115]	UFPR	Micro	112/2240	SVM	80.7	88.5	91.5
Paula Filho et al. [62]	UFPR	Macro	41/2942	SVM	56.0	68.2	61.8
Martins et al. [95]	UFPR	Micro	112/2240	SVM	4.1	66.3	86.7
Yadav et al. [122]	UFPR	Micro	75/1500	SVM	–	79.9	93.5
da Silva et al. [71]	RMCA	Micro	77/1221	*k*-NN (*k* = 1)	–	85.0	87.4

*Stereo* stereogram, *Macro* macroscopic image, *Micro* micrograph, *XCT* X-ray computed tomographic image, *#SP* number of species, *#IMG* number of images, *ANN* artificial neural network, *SVM* support vector machine, *k-NN* *k*-nearest neighbors

Local phase quantization (LPQ) is an algorithm for extracting blur insensitive textures using Fourier phase information. As shown in Table 3, LPQ was applied primarily to micrographs, where it performed better than GLCM and LBP [71, 95, 115, 122]. One study has investigated LPQ on macroscopic scale image datasets, but the discriminative power of LPQ was lower than that of LBP in a comparative study using the UFPR macroscopic image dataset [62].

In addition to the textures described above, other texture features such as higher local order autocorrelation (HLAC) and Gabor filter-based features have been used for wood classification, and because of their high classification accuracy they have proved to be promising feature extractors for wood identification [68, 101, 123–125]. Texture fusion strategies for different types of texture features have always been superior to a single feature set in terms of classification accuracy [28, 80, 115].

#### Local feature

Local features are distinct structural elements such as points, corners, and edges in an image, vs. textures. The biggest difference between local features and textures is that textures are descriptors that describe an image as a whole, whereas local features describe interesting or important local regions called keypoints. That is, the texture feature is an image descriptor and the local feature is a keypoint descriptor. Local feature extraction thus consists of two major processes, feature detection and feature description. In general, local features are better at handling image rotation, scale, and affine changes [18, 19].

Scale-invariant feature transform (SIFT) developed by Lowe in 2004 [18], is a local feature extraction algorithm that has been the benchmark for local feature collection in CV since its introduction. SIFT detects blobs, corners, and edges as keypoints in an image and represents local regions of the image as 128-dimensional vectors calculated based on the gradient orientations of the pixels around each keypoint.

Most of the local features used for wood identification have been collected using SIFT [48, 54, 73, 79, 93]. Hwang et al. [73] investigated the discriminative power of SIFT for image resolution using the XDD Lauraceae micrograph dataset. Taking into account the identification accuracy and the computational cost, they reported that a pixel resolution of about 3 µm was appropriate for wood identification. If the image was shrunk beyond the 3-µm pixel resolution, the accuracy was greatly reduced because information about the wood fibers was lost. However, from an identification study of Fagaceae species, Kobayashi et al. [48] reported that a satisfactory identification performance could be obtained at a pixel resolution of 4.4 µm, even though some of the wood fiber information was lost.

Hwang et al. [74] compared the wood identification performance of each model trained with well-known local feature extraction algorithms: SIFT, speeded up robust features (SURF) [19], oriented features-from-accelerated-segment-test (FAST), rotated binary-robust-independent-elementary-feature (BRIEF) (ORB) [126], and accelerated-KAZE (AKAZE) [127]. Without considering the computational cost, SIFT had the highest discriminative power among the algorithms tested (Table 4). Visualization of the features extracted by each algorithm confirmed that SIFT detected cell corners more effectively than the other algorithms. Hwang et al. [74] noted that in cross-sectional images of wood, the cell corner is an important feature for identification because it contains information about the mode of aggregation of different cell elements. The superiority of SIFT seems to be because it was designed to detect corners.

**Table 4:** Performances of major local features and textures for wood identification

References	Database	Image type	#SP/#IMG	CLS	Classification rate (%)						
					Local features				Textures		
					SIFT	SURF	ORB	AKAZE	GLCM	LBP	LPQ
Hu et al. [128]	–	Macro	28/2800	ANN	90.2	–	–	–	63.8	85.7	–
Martins et al. [47]	UFPR	Micro	112/2240	SVM	88.5	89.1	–	–	4.1	66.3	86.7
Hwang et al. [74]^a^	XDD^b^	Micro	9/1019	SVM	79.2	42.2	63.6	61.6	–	–	–

*Macro* macroscopic image, *Micro* micrograph, *#SP* number of species, *#IMG* number of images, *CLS* classifier, *ANN* artificial neural network, *SVM* support vector machine

^a^F1 score was used as a performance metric

^b^Lauraceae wood dataset in the XDD database

In comparative studies of local features and textures (Table 4), SIFT and SURF had higher discriminative power than GLCM and LBP, whereas LPQ had similar discriminative power for local features [47, 128]. Histograms of oriented gradients (HOG) [129] are descriptors that represent a local region of an image, and they have been used to classify macroscopic image datasets [130].

Describing a whole image using only local features can limit the classification of images with complex structural elements. To solve this problem, a codebook-based framework has been used to quantify the extracted features. The bag-of-features (BOF) model, which uses codewords generated by the clustering of extracted features, is an effective method of quantifying features to represent an image [131]. The BOF model trained with codewords converted from SIFT descriptors produced higher classification performance than the model trained with SIFT features intact [93]. Even for a macro image dataset, the SIFT-based BOF model outperformed the models trained with texture features [128].

#### Other features

In addition to the features described above, other feature types such as color and anatomical statistic features have been used for hardwood identification. Such features were used mainly in combination with other types of features because their discriminative power as a single feature set was relatively inadequate, and multiple feature set strategies that combined different types of features produced improved results for identification accuracy [58, 62, 77].

Color is the most intuitive feature for human vision, but it is unstable as a feature. Wood color is not only variable by environmental factors such as tree growth conditions and atmospheric exposure time, but also by variability in an individual tree such as heartwood and sapwood, and earlywood and latewood. Wood identification has been carried out based on color differences between species, but large intra-species color variations are a big obstacle. Zhao et al. [63, 78] proposed novel color recognition systems that efficiently distinguished between intra- and inter-species color variations using an improved snake model [132] and an active shape model [133] with a two-dimensional image measurement machine. They also built a classification model that outperformed their previous models using a fusion scheme of color, texture, and spectral features [116]. Color features have primarily been used in combination with textures as part of macro level multi-feature sets to improve discrimination [58, 62, 77, 134].

Using image segmentation techniques, anatomical statistical features such as the shape, size, number, and distribution of specific wood cells can be extracted from a cross-sectional image. The vessel is the most characteristic anatomical element in macroscale images, so it was particularly preferred for statistical feature extraction. Yusof et al. [28, 59] extracted the statistical properties of pore distribution (SPPD) from the FRIM database and used them as features. Pre-classification of the SPPD features based on fuzzy logic [135] improved the accuracy of the identification system and reduced the processing time. Other similar studies have successfully used statistical features to identify wood [61, 80, 103].

### Dimensionality reduction and feature selection

Large numbers of extracted features reduce the computational efficiency of classification models, therefore it is important to find a balance between classification accuracy and computational cost. Principal component analysis (PCA) [136] and linear discriminant analysis (LDA) [107] are representative methods for dimensionality reduction of data. da Silva et al. [71] reduced the dimensionality of LPQ and LBP features extracted from the RMCA database using PCA and LDA. Their classification model produced promising results, even though the feature data were significantly reduced. Kobayashi et al. [48] reduced the 128-dimensional SIFT feature vectors extracted from Fagaceae micrographs to 17 dimensions using LDA, and the wood identification using the reduced feature set was quite accurate.

Whereas dimensionality reduction converts image features into new numerical features, feature selection takes the essence of the features without converting them. The genetic algorithm (GA) is an engineering model that borrows from the biological genetic and evolution mechanisms. The GA finds a better solution by repeating the cycle of feature selection, crossover, mutation, evaluation, and update [137]. Khairuddin et al. [138] applied the GA to texture features extracted from the FRIM database and used the selected features to train their model. They found that the model performance was 10% more accurate than the same model in a previous study [26], even though the GA reduced the dimensionality of the training data by half. Yusof et al. [28] further improved the feature selection by combining the GA with kernel discriminant analysis. Another feature selection algorithm is the Boruta algorithm, which selects key variables based on Z-score using random forest [139].

### Classification

Classifiers create classification models by learning the features extracted from a training set and establishing classification rules. The training phase ends with the implementation of the classification model. In the test phase, image features are extracted from the test set through the same processes as the training set. The features are then entered into the classification model and the model classifies each image, which completes the classification of the test set.

The three most preferred classifiers in the wood identification studies were *k*-nearest neighbors (*k*-NN) [140], support vector machine (SVM) [141], and artificial neural network (ANN) [142, 143]. *k*-NN is the simplest ML algorithm, which simply stored the training data and focuses on classifying query data. To classify query data, the algorithm finds *k* data points closest to the target data point in the training dataset based on the Euclidean distance [140]. Minimum distance-based classifiers [140, 144] such as *k*-NN work best on small datasets because the amount of required system space increases exponentially as the number of input features increases [145].

SVM is an algorithm that clearly classifies data points in *N*-dimensional space by finding a hyperplane with the maximum margin between classes of data points [141]. Basically, SVM is a linear model, but combining it with kernel methods enables nonlinear classification by mapping data into higher dimensional feature spaces [146]. SVM requires less space than *k*-NN because it learns the training data and builds a classification model in advance. SVM has been shown to outperform *k*-NN for wood identification [68, 73, 128, 147, 148]. In most studies that compared the two classifiers in the same classification strategy, SVM outperformed *k*-NN (Table 5). Because the SVM algorithm, as well as other ML algorithms, is very sensitive to the parameters, gamma (or sigma), a Gaussian kernel parameter for nonlinear classification, and cost (*C*), a parameter that controls the cost of misclassification on the training data [149], parameter optimization using techniques such as grid search or GA is essential [150, 151].

**Table 5:** Performances of *k*-NN, SVM, and ANN classifiers reported in CV-based wood identification studies

References	Database	Image type	#SP/#IMG	Feature	Classification rate (%)		
					*k*-NN	SVM^a^	ANN
Hu et al. [128]	–	Macro	28/2800	SIFT	77.3	87.5	90.2
Tou et al. [117]	CAIRO	Stereo	5/500	GLCM	63.6	–	72.8
Prasetiyo et al. [147]	FRIM	Stereo	25/2390	LBP	80.0	85.6	93.6
Wang et al. [148]	ZAFU WS 24	Stereo	24/480	GLCM	87.5	91.7	–
Wang et al. [68]	ZAFU WS 24	Stereo	24/481	HLAC with MMI	76.3	87.7	–
Souza et al. [52]	–	Stereo	64/1901	LBP	–	98.1	96.5
Yadav et al. [192]	UFPR	Micro	25/500	1st Stat. with Coiflet DWT	–	65.2	92.2
Hwang et al. [73]	XDD	Micro	39/1557	SIFT	84.3	95.4	–
Kobayashi et al. [48]	XDD	Micro	18/2406	SIFT, CC	95.3	93.1	–

*#SP* number of species, *#IMG* number of images, *k-NN* *k*-nearest neighbors, *SVM* support vector machine, *ANN* artificial neural network, *Stereo* stereogram, *Micro* micrograph, *Macro* macroscopic image, *MMI* mask matching image, *1st Stat* first order statistic features, *DWT* discrete wavelet transform, *CC* connected component labelling

^a^Linear kernel SVM classifier

The ANN algorithm mimics the learning process of the human brain and is the foundation of DL, which is the mainstream of modern computer science [152–155]. This algorithm has produced state-of-the-art performance as a classifier in wood identification as well as in various other classification problems. Regardless of the type of image and feature, ANN performed better than *k*-NN and SVM (Table 5). Wood features are nonlinear relationships, and ANN is able to learn such complicated nonlinear relationships [156]. In addition, ANNs are resilient in the face of noise and unintentional features in the data [106]. The ANN algorithm is a backpropagation algorithm that refines the model by updating the weights through propagation of the prediction error to previous layers. [157]. These characteristics make it suitable for wood identification. ANN has been used with large datasets [72, 97, 128], which supports DL methods such as CNN that are able to build more sophisticated models with larger amounts of data [53, 55, 64, 96].

Although ANN is a good classifier, there is no one optimal classifier for wood identification. For new datasets, it has been recommended that it is better to start with a simple model such as *k*-NN to develop an understanding of the data characteristics before moving to a more complex model such as SVM or ANN [158]. Depending on the purpose, ensembles of multiple classifiers also may be considered [53, 64, 93].

## Deep learning

### Convolutional neural networks

**Fig. 5:**
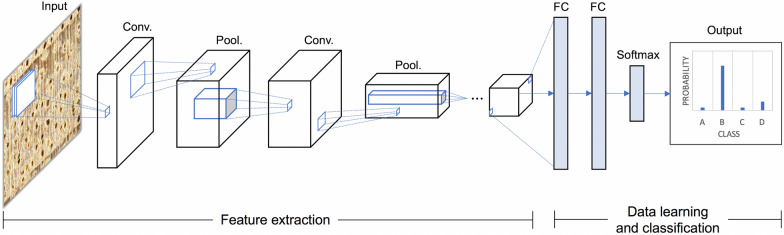
Typical simple convolutional neural network architecture. *Conv* convolution unit, *Pool* pooling unit, *FC* fully-connected layer

Convolutional neural networks (CNN) were first introduced to process images more effectively by applying filtering techniques to artificial neural networks [159]. Afterward, a modern CNN framework for DL was proposed by LeCun et al. [17]. Typical CNN architecture is shown in Fig. 5. There are many layers between input and output. Convolution and pooling layers extract features from images, and fully-connected layers are neural networks that learn features and classify images. In a convolution layer, a feature map is generated by applying a convolution filter to the input image. In a pooling layer, only the important information is extracted from the feature map and used as input to the next convolution unit. Convolution filters can start with very simple features, such as edges, and evolve into more specific features of objects, such as shapes [46]. Features extracted from the convolution and pooling layers are passed to the fully-connected layers and then classification is performed by a deep neural network.

A deep neural network is composed of several layers stacked in a row. A layer has units and is connected by weights to the units of the previous layer. The neural network finds the combinations of weights for each layer needed to make an accurate prediction. The process of finding the weights is said to be training the network. During the training process, a batch of images (the entire dataset or a subset of the data set divided by equal size) is passed to the network and the output is compared to the answer. The prediction error propagates backward through the network and the weights are modified to improve prediction [157]. Each unit gradually becomes equipped with the ability to distinguish certain features and ultimately helps to make better predictions [46].

### CNN in wood identification

Table 6 lists wood identification studies using CNN models. All studies have been reported within the last decade and are accelerating over time. Hafemann et al. [29] used a CNN model combined with an image patch extraction strategy to classify macroscopic image and micrograph datasets in the UFPR database. The classification performance of the CNN model outperformed those of models trained with texture features. Notably, their CNN model was designed with only two convolution units. Kwon et al. [64, 96] successfully classified six Korean softwood species using CNN-based models, LeNet [17], mini VGGNET [160], and their ensemble models, which have been successful in general image classification. Oliveira et al. [161] developed a wood identification software based on CNN models trained with the UFPR database. The architecture of their CNN models was not disclosed, but the model that they chose as the basis for the software performed best performance in studies of both macroscopic and micrograph datasets using the UFPR database as a benchmark.

**Table 6:** Performances of deep learning models reported in CV-based wood identification studies

References	Dataset	Image type	#SP/#IMG	CNN model	CLS %
Hafemann et al. [29]	UFPR	Macro	41/2942	3-ConvNet^a^	95.8
		Micro	112/2240	3-ConvNet^a^	97.3
Kwon et al. [96]	Softwoods	Macro	5/16,865	LeNet	99.3
Kwon et al. [64]	Softwoods	Macro	5/33,815	Ensemble of LeNet2, LeNet3, and MiniVGG4	0.98^b^
Ravindran et al. [81]	Meliaceae species	Stereo	10/2303	VGG16	88.7^c^
					97.5^d^
Tang et al. [49]	FRIM collection	Stereo	100/101,446	SqueezeNet	77.5
Lopes et al. [51]	FWRC collection	Stereo	10/1869	InceptionV4_ResNetV2	92.6
Lens et al. [72]	UFPR	Micro	112/2240	ResNet101	96.4
de Geus et al. [55]	Brazilian species	Stereo	281/–	DenseNet	98.8
Ravindran [21]	Melaceae species	Stereo	10/–^e^	ResNet34	81.9
					96.1
Fabijanska et al. [65]	European species	Macro	14/312	Residual convolutional encoder network	98.7

#SP: number of species; #IMG: number of images; CLS %: classification accuracy; FWRC: Forest and Wildlife Research Center at Mississippi State University; –: no number specified

^a^3 layers deep CNN

^b^F1 score

^c^Species identification

^d^Genus identification

^e^At least 5 images per specimen (total 193 wood specimens)

CNNs generally require large databases. However, large wood image databases with the correct labels are quite difficult to obtain. To expect competitive performances from CNN-based models, they are known to require a database that is at least 10 times larger than that required for feature engineering-based methods [91]. Therefore, transfer learning was introduced as a network training method for small databases [162]. Transfer learning provides a path to building competitive models using a moderate amount of data by leveraging pre-trained networks with the ImageNet dataset [163].

Ravindran et al. [81] classified 10 neotropical species with high accuracy using the VGG16 [160] model with transfer learning. Tang et al. [49] developed a smartphone-based portable macroscopic wood identification system based on the SqueezeNet [164] model. In a comparative study of DL and conventional ML models [55], CNN-based models, Inception-v3 [165], SqueezeNet [164], ResNet [25], and DenseNet [166], all models achieved better performance than k-NN models trained with LBP or LPQ features. Lens et al. [72] also reported that VGG16 and ResNet101 models had better classification performance for the UFPR dataset than those trained with texture features. The CNN with residual connections proposed by Fabijańska et al. [65] identified 14 European trees better than other popular CNN architectures.

## Field-deployable wood identification systems

Many studies have aimed to develop CV-based automated wood identification systems, and some have been realized as field-deployable systems [21, 49, 51, 69]. XyloTron, developed by the Forest Products Laboratory, US Department of Agriculture, is a CNN-based wood identification system using ResNet34 backbone [21]. This is the only system to report actual field use in real-time and showed higher species and genus identification performance for the family Meliaceae than the mass spectrometry-based model [21].

Recently, smartphone-based systems have been developed that require minimal components. MyWood-ID can identify 100 Malaysian woods [49], and another smartphone-based system, AIKO, can identify major Indonesian commercial woods [83]. XyloPhone, a smartphone-based imaging platform, has been introduced as a field-use identification tool that is more affordable and scalable than other commercial products [57]. All the field-deployable systems are based on stereogram datasets [21, 49, 51, 57, 69, 83].

## New aspects in wood science: CV-based wood anatomy

From the outset, CV-based wood identification was an informatics-driven research field, so most studies were focused only on improving the identification performance, and not on the wood itself. With the recent interest in AI, new aspects have emerged to understand CV based on the domain knowledge of wood science.

### Feature-based anatomical approaches

Kobayashi et al. [99] conducted PCA on GLCM features extracted from hardwood stereograms to investigate the relationship between anatomical structures and texture features. From principal component loadings and analysis of macroscopic patterns of wood they found that each Haralick texture feature correlated with different anatomical structures such as vessel population, ray-to-ray spacing, and tylosis abundance.

A *k*-means clustering [167] analysis of local features extracted from micrographs suggested the possibility of matching feature clusters with anatomical elements [73]. This idea was extended to quantify anatomical elements by encoding local features into codewords. The BOF framework effectively visualized and assigned local feature-based codewords to anatomical elements of wood, and codeword histograms provided an indirect means of quantitative wood anatomy [74]. The finding that local features are the vehicles for access to CV from an anatomical point of view is the main reason that much attention is being paid to the applications of informatics in the wood science field.

**Fig. 6:**
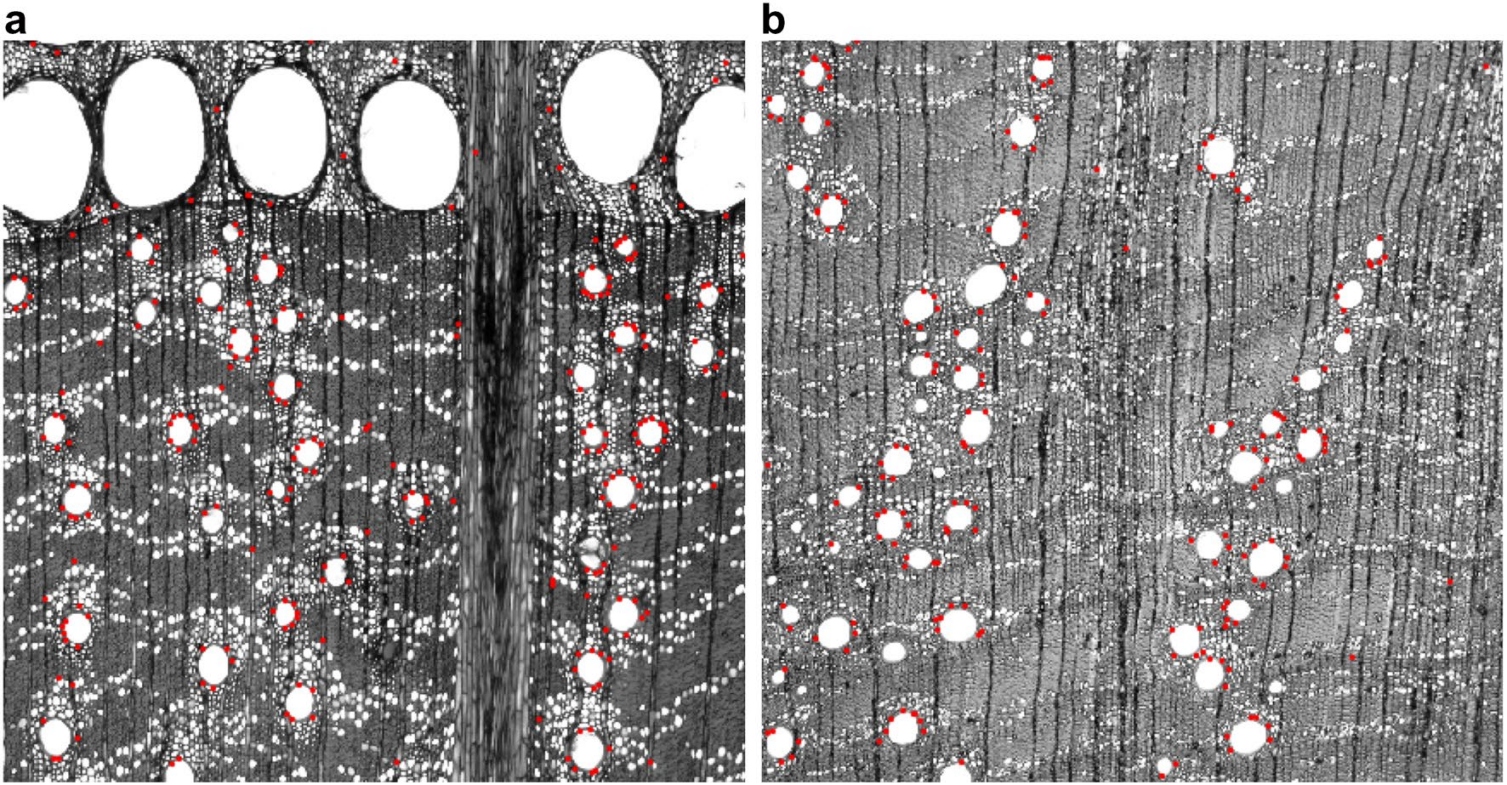
Taxon-specific features based on keypoint clustering of a Fagaceae micrograph dataset with SIFT features. Keypoints (red dots) commonly occurred in both *Quercus acutissima* (Cerris) (**a**) and *Q. phillyraeoides* (IIex) (**b**). Figure copyright (Kobayashi et al.), licensed under CC-BY 3.0

Hierarchical clustering of a Fagaceae micrograph dataset with SIFT features confirmed that the clustering basically coincided with wood porosity. Moreover, the relationship of species groups that contradicted wood porosity was consistent with evolution based on molecular phylogeny [48]. An unexpected finding was that the subgenera Cerris (ring porous wood) and Ilex (diffuse porous wood) had common characteristics in the arrangement of the latewood vessels as predicted by a CV-based analysis (Fig. 6).

### Feature importance measures

For promising results produced by established wood identification models, the features that contributed to the identification need to be assessed. Random forest [168], an ensemble algorithm and classification model that combines multiple decision trees [169], may provide an effective means to do this. This algorithm has been preferred in bioinformatics and biological data-based studies because it provides variable importance, which is the feature importance that evaluates and ranks the variables for the predictive power of a model [170–172]. Feature importance measures can be used to interpret the identification results produced by a model into domain knowledge of wood anatomy.

**Fig. 7:**
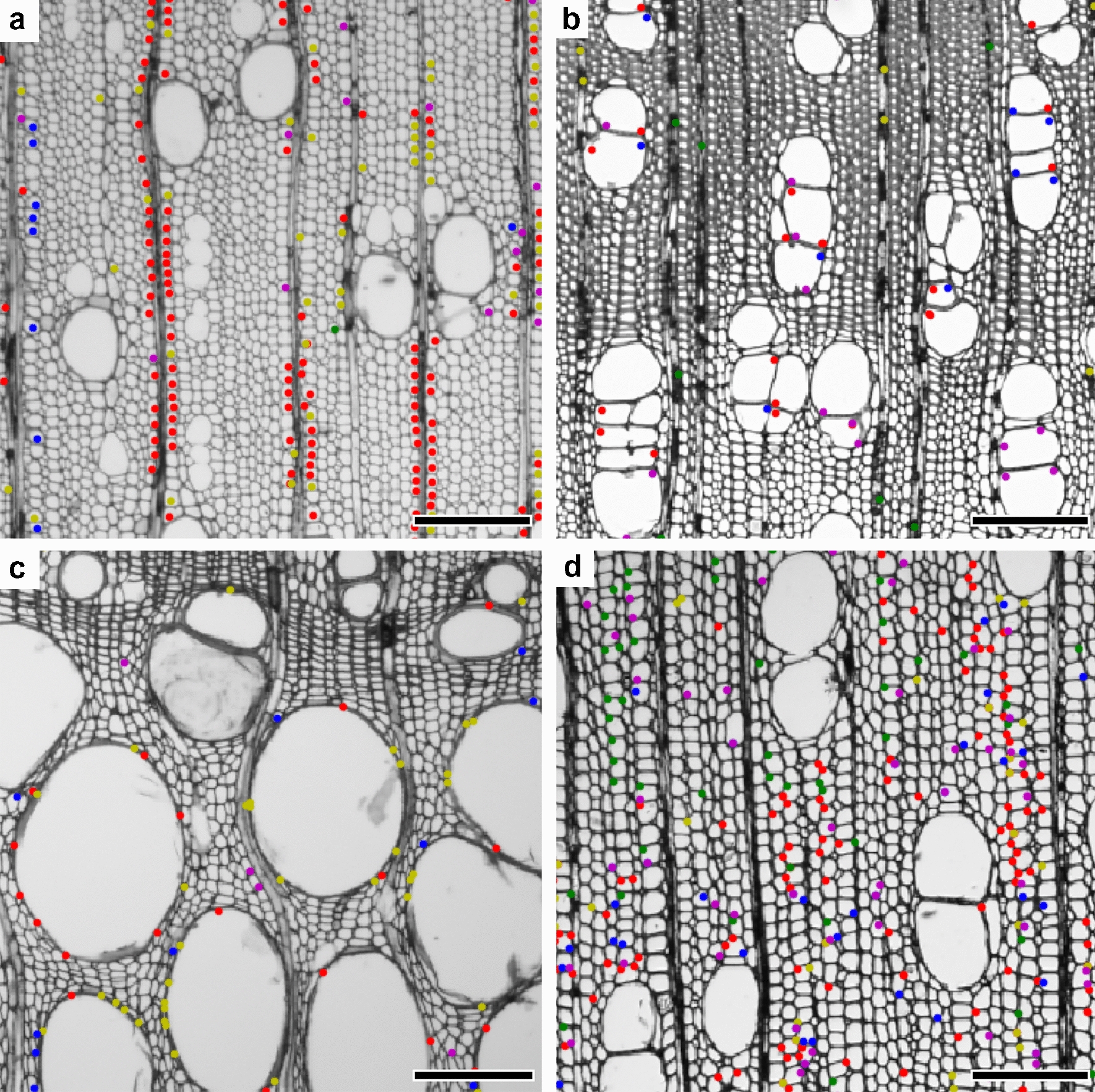
Visualization of informative features with the highest TFIDF scores from the Lauraceae micrograph dataset. **a** Wood fibers adjacent to rays in *Machilus pingii*. **b** Intervessel walls in *Lindera communis*. **c** Large vessels in *Sassafras tzumu*. **d** Wood fibers in earlywood in *Phoebe macrocarpa*. The frequency–inverse document frequency (TFIDF) scores run from high to low in the order red, yellow, green, blue, and purple. Scale bars = 200 µm

Another feature importance measurement technique using codewords is the term frequency–inverse document frequency (TFIDF) score [173]. TFIDF is derived from bag-of-words (BOW) [174], a model for document retrieval and the origin of BOF, and the TFIDF score provides keywords for document retrieval. Similarly, the BOF model uses image features and provides informative features for image classification. A codeword with a high TFIDF score indicates a rare feature present in a small number of species, whereas a low score denotes a feature shared by many species [74]. As shown in Fig. 7, the features with high TFIDF scores for each species are different, and can be used to infer species-specific features.

In CNN models, the class activation map (CAM) shows discriminative image regions that contribute to classifying images into specific classes [175, 176]. To produce a CAM, the model classifies images by performing global average pooling on feature maps in the last convolution layer, and then regions of interest are detected from the weights and the feature maps. Nakajima et al. [177] used a CAM to analyze important image regions for the dating of each annual ring in tree ring analysis.

### Cell segmentation using deep learning

The anatomical composition of wood cells reflects environmental changes during the growth period of the tree [178, 179], therefore analysis of cell variability, that is, quantitative wood anatomy, helps answer questions related to tree functioning, growth, and environment [180]. The laborious and tedious task of identifying and measuring hundreds of thousands of wood cells is a major obstacle to do it. Several studies have been reported for automated segmentation of wood cells using classical image processing techniques [75, 179, 181], but the results were highly dependent on image quality and manual editing by the operator.

With recent advances in CV and DL technologies, CNNs have shown remarkable achievements in segmenting cells from biomedical microscopic images [155, 182, 183]. The state-of-the-art techniques are also been applied to the segmentation of plant cells, including wood [184, 185]. Garcia-Pedrero et al. [185] segmented xylem vessels from cross-sectional micrographs using Unet [155], a multi-scale encoder-decoder model based on CNN. They reported that the vessel segmentation by Unet was closer to the results of the expert’s work using the image analysis tool ROXAS [186] than the classical techniques Otsu’s thresholding methods [187] and morphological active contour method [188]. In a comparative study of three of the latest neural network models, Unet, Linknet [189], and Feature Pyramid Network (FPN) [190] for vessel segmentation, the models had a high pixel accuracy of about 90% as well as a shorter working time than the existing image analysis tool [186].

The segmentation results produced from CNN-based models demonstrate the potential of DL to perform quantitative wood anatomy more effectively, overcoming obstacles such as the non-homogeneous illumination or staining of images, where conventional methods tend to yield unsatisfactory results [191]. DL is evolving rapidly and has provided excellent results in many fields of study. This can provide solutions to the questions of wood identification and anatomy and is an opportunity and challenge to bring new insights into wood science.

## Conclusions

CV-based wood identification continues to evolve in the development of on-site wood identification systems that enable consistent judgment without human prejudice. Furthermore, by allowing an anatomical approach, CV-based wood identification may provide insights that have not yet been revealed by established wood anatomy methods. The first and most important task to progress CV-based wood identification is to build large digital databases where wood information can be accessed anytime, anywhere, and by anyone, and to bridge the current gap between informatics and wood science.

DL in recent years has provided a technical foundation for more accurate wood identification and is expected to answer a variety of questions in wood anatomy shortly. Advances in communication technologies provide a broad space for the use of CV-based wood identification in the field. This could ultimately be a solution to the on-site demands by allowing wood identification by workers who are not trained in traditional identification. From the macro perspective of modern science, it is clear that CV, ML, and DL technologies will contribute to the development of the various subfields encompassed by wood science.

## Data Availability

Not applicable.

## Abbreviations

AI: Artificial intelligence
AKAZE: Accelerated-KAZE
ANN: Artificial neural network
BGLAM: Basic gray level aura matrix
BOF: Bag-of-features
BOW: Bag-of-words
BRIEF: Binary-robust-independent-elementary-feature
CAM: Class activation map
CNN: Convolutional neural network
CT: Computed tomography
CV: Computer vision
DL: Deep learning
FAST: Features-from-accelerated-segment-test
GA: Genetic algorithm
GLCM: Gray level co-occurrence matrix
HLAC: Higher local order autocorrelation
HOG: Histograms of oriented gradients
IAWA: International Association of Wood Anatomists
*k*-NN: *k*-Nearest neighbors
LBP: Local binary pattern
LDA: Linear discriminant analysis
LPQ: Local phase quantization
ML: Machine learning
ORB: Oriented FAST and rotated BRIEF
PCA: Principal component analysis
SIFT: Scale-invariant feature transform
SPPD: Statistical properties of pores distribution
SURF: Speeded up robust features
SVM: Support vector machine
TFIDF: The term frequency–inverse document frequency
XCT: X-ray computed tomography
